# An Erbium-Based Bifuctional Heterogeneous Catalyst: A Cooperative Route Towards C-C Bond Formation

**DOI:** 10.3390/molecules190710218

**Published:** 2014-07-15

**Authors:** Manuela Oliverio, Paola Costanzo, Anastasia Macario, Giuseppina De Luca, Monica Nardi, Antonio Procopio

**Affiliations:** 1 Dipartimento di Scienze della Salute, Università Magna Graecia, Viale Europa, 88100 Germaneto (CZ), Italy; E-Mails: pcostanzo@unicz.it (P.C.); procopio@unicz.it (A.P.); 2 Dipartimento di Ingegneria per l’Ambiente e il Territorio e Ingegneria Chimica, Università della Calabria, 87036 Arcavacata di Rende (CS), Italy; E-Mail: anastasia.macario@unical.it; 3 Dipartimento di Chimica, Università della Calabria, Cubo 12C, 87036 Arcavacata di Rende (CS), Italy; E-Mails: g.deluca@unical.it (G.D.L.); monica.nardi@unical.it (M.N.)

**Keywords:** aldol reaction, bifunctional catalysis, Henry reaction, lanthanides, mesoporous silica

## Abstract

Heterogeneous bifuctional catalysts are multifunctional synthetic catalysts enabling efficient organic transformations by exploiting two opposite functionalities without mutual destruction. In this paper we report the first Er(III)-based metallorganic heterogeneous catalyst, synthesized by post-calcination MW-assisted grafting and modification of the natural aminoacid L-cysteine. The natural acid–base distance between sites was maintained to assure the cooperation. The applicability of this new bifunctional heterogeneous catalyst to C-C bond formation and the supposed mechanisms of action are discussed as well.

## 1. Introduction

Cooperation between different functionalities on the same catalytic system is a biological strategy for efficient organic synthesis. Enzymes are the main example of multifunctional catalysis in Nature. Among them, metalloenzymes are the subset employing organic functional groups in cooperation with metal ions, working as Lewis acids or redox centers, for the contemporary activation of nucleophiles and electrophiles [1]. This natural strategy has inspired chemists to design bifunctional catalysts, many of which combine a metal ion, usually as part of a chiral Lewis acid complex, with an organic function working as a Lewis base [2]. Acids and bases are antagonists, so that the main problem associated with their cooperation in homogeneous phase in the same reactor, is their mutual neutralization. A common strategy to overcome the self-quenching problem is to combine the right pair of base and metal ion, relying on the hard-soft theory stating that a hard metal ion is not affected by a soft organic base [1,3]. Thus realizing a heterogeneous system, supporting the catalyst on a solid matrix, has been recently proposed as a route to overcome the mutual destruction of acid and basic sites by providing a positioning control at an appropriate distance for cooperation [4,5,6,7,8,9,10]. The site isolation and the equivalent mole relationship between acid and base sites exploiting a single functionalizing molecule bearing the two functionalities in a protected form, such as an aminoacid, has been recently achieved [11]. Heterogeneous bifuctional materials were often prepared by co-condensation. To the best of our knowledge only few reports exist concerning the post–calcination grafting of multifunctional agents on the surface of mesoporous silica [12], many of them related to the immobilization of expensive chiral ligands for heterogeneous Lewis acid complexes [13,14].

Our studies started from the observation that Er^III^ has several advatageous features with a view to the design of a new metallorganic heterogeneous catalyst: its low Price, which is due to its wide application in telecomunications [15]; the low toxicity of its salts [16,17,18]; its small radius that contributes to its high oxyphilicity and Lewis acidity [19]. Very recently Tiseni and Peters demonstrated that Er(OTf)_3_ can cooperate with normal bases in homogeneous phase, without self-quenching [20]. Moreover, in recent years we have developed good skills in applying microwave heating to the grafting of different organic moieties on the surface of the mesoporous silica [21] and we successfully utilized this procedure to obtain a new hybrid mesoporous silica–supported Er^III^ catalyst. We demonstrated that this catalyst is very efficient in a wide series of common organic transformation involving the C-C bond formation, protection and deprotection of alcohols and carbonyl compounds [22,23,24].

## 2. Results and Discussion

**Scheme 1:**
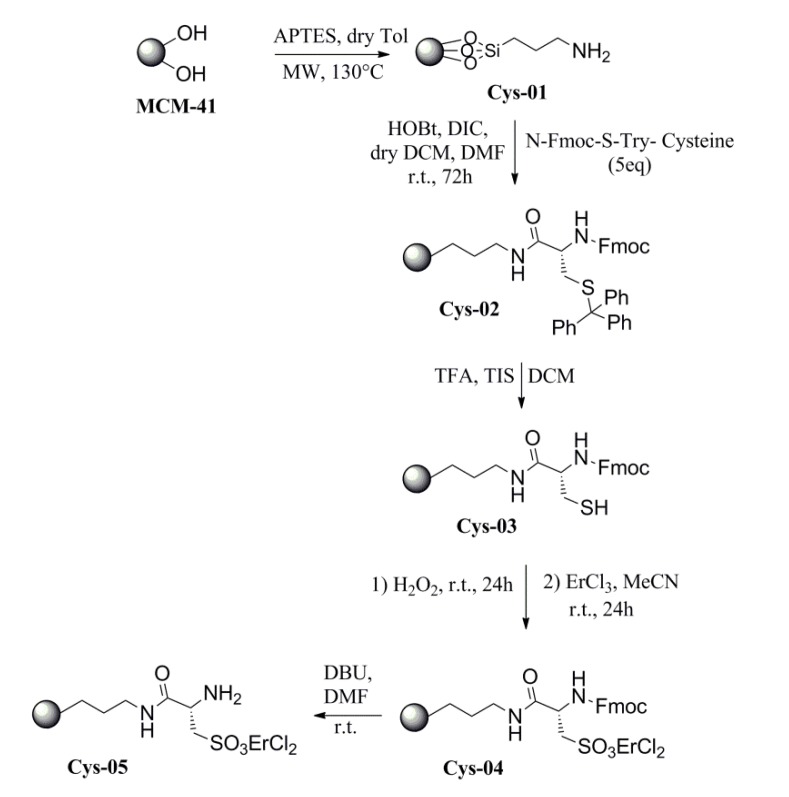
Synthesis of Er-Cys05.

Starting from this background, we designed a bifunctional catalyst where ErCl_3_ was coordinated to the mesoporous surface of MCM-41 silica, using the natural aminoacid L-cysteine (Scheme 1) as organic moiety. The L-cysteine, after grafting and modification of the lateral thiolic chain, can offer the sulfonyl Er^III^ ligand, and, at the same time, the primary amino group working as cooperative base. This strategy allows not only to preserve the natural distance existing between the amino-terminal group and the lateral chain of the aminoacid, but even to ensure the equivalent mole relationship between the metal center and the amino function. The full synthetic pathway is depicted in Scheme 1.

**Figure 1:**
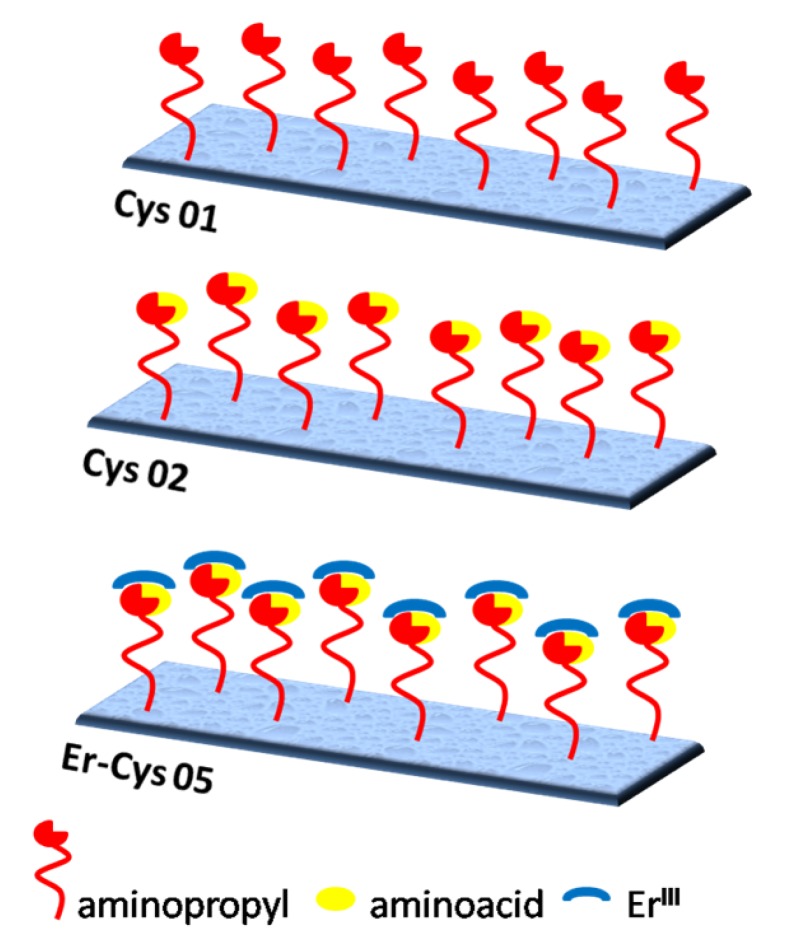
Schematic representation of Er-Cys05 catalyst.

The synthesis started from the MW-assisted grafting of an aminopropyl moiety on the silica surface following the protocol already published [21]. The N-Fmoc-S-Trt-L-cysteine coupling was realized on the aminopropyl chain in presence of DIC and HOBt, according to the “active ester” method [25]. The deprotection of the thiolic moiety [26] was followed by its oxidation to sulfonyl function in presence of H_2_O_2_ The coordination of Er^III^ was achieved by stirring the solid in a suspension of ErCl_3_ in refluxing CH_3_CN [22,23]. The cleavage of Fmoc–protecting group gave rise to the final catalyst Er-Cys05 (see Supplementary Materials). Er-Cys05 had a high loading of organic spacers on its surface (1.00 mmol/gr of silica) completely covered by the aminoacid and the metal center Er^III^ (Figure 1).

**Table:** 

	Total [-NH_2_] (mmol/gr)	AA [-NH_2_] (mmol/gr)	[Er^m^] (mmol/gr)
**Cys 01**	1.00	1.00	-
**Cys 02**	1.00	1.00	-
**Er-Cys05**	0.94	0.94	0.94

Table 1 summarizes the most important characterization data of all the synthesized materials.

**Table 1:** Material characterization summary.

Entry	Compound	Total [-NH_2_] (mmol/gr)	[Er^III^] (mmol/gr)	Textural Properties			FT–IR 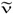 (cm^−1^)
				SBET (m^2^/gr)	BJH Pore Volume (cm^3^/gr)	BJH Average Pore Diameter (Å)	
**1**	MCM-41	-	-	1600	1.70	35	452 (s, *νs* (Si-OSi)), 3458 (*vs*, *νs* (HO-H))
**2**	Cys 01	1.00	-	Ref30 ^a^	Ref30 ^a^	Ref30 ^a^	461 (s, *νs* (Si-OSi)), 1630 (b, N-H), 2938 (*vs* C-H), 3446 (*vs*, *νs* (HO-H))
**3**	Cys 02	1.00	-	-	-	-	-
**4**	Cys 03	0.94	-	-	-	-	-
**5**	Er-Cys 04	0.94	0.94	-	-	-	-
**6**	Er-Cys05	0.94	0.94	327	0.24	40	465 (s, *νs* (Si-OSi)), 791 (m, *νs* (C-S)), 1312 (w, *νs* (S=O)), 1550 (w, δ (N-H/C-N)), 1650 (s, *νs* (C=O)), 2342 (w, *νs* (N-H_3_^+^)), 3442 (vs, *νs* (HO–H))

^a^ For the textural properties of this material see reference [27].

We evaluated the amino-loading on the silica by the spectrophotometric analysis of the Fmoc-protecting groups. The Er^III^ loading was measured by ICP-MS. The success of the aminoacid coupling was proved by ^13^C-NMR and FT-IR measurements on the silica that clearly revealed the formation of the peptide bond with a characteristic signal at 1451 cm^−1^ (see Supplementary Materials).

### Characteristics of Catalyst Surface were Monitored by N_2_ Adsorption-Desorption Technique

**Figure 2:**
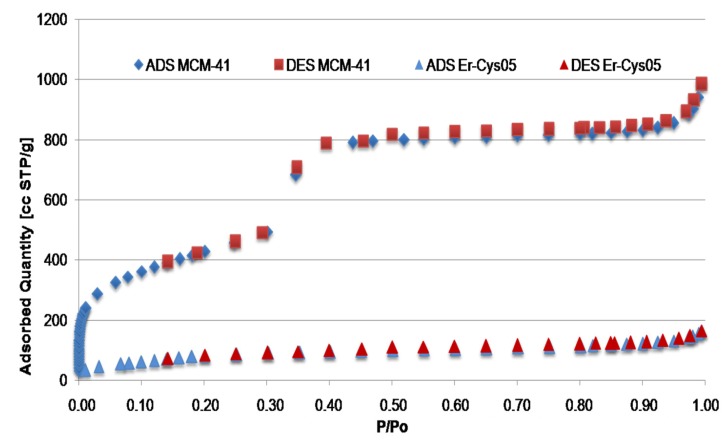
N_2_ adsorption isotherms.

As expected, the catalyst shows a lower specific surface area with respect to the starting MCM-41 support, after loading of active sites (Figure 2). After grafting, the N_2_ adsorption isotherm of Er-Cys05 material shows that this catalyst preserves a slight regular mesoporosity, even if the meniscus part of the curve is broader and occurs at lower relative pressure with respect to the isotherm of the starting support (see Supplementary Materials).

**Scheme 2:**
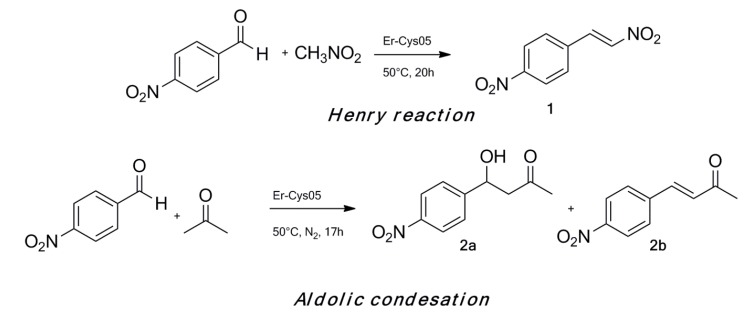
General synthetic scheme for Henry reaction and aldol condensation.

In order to investigate how the intrinsic cooperation between the acid metal center and the amino group works, we decided to test the bifunctional catalyst in the Henry reaction and the aldol condensation (Scheme 2, see Supplementary Materials). It has been reported that cooperation between acids and bases on the same support strongly enhance the efficiency and rate of both the reactions [28,29] depending on the molar relationship between sites and the specific amine – primary, secondary and tertiary amines giving deeply different results [4,13,28,29].

**Table:** 

Catalyst	S BET (m^2^/gr)	Pore Volume (cm^3^/gr)	Average Pore Diameter (Å)
**MCM-41**	1600	1.70	35
**Er-Cys05**	330	0.24	40

We performed a comparative study (Table 2) of the bifuctional catalyst, the heterogeneous acid Lewis catalyst MCM-Er and the aminopropyl silica MCM-NH_2_, prepared as previously reported [21].

TON (turnover number) and TOF (turnover frequency) for all used catalysts were determined at the end of every catalytic cycle as described in Table 2. The cooperative effect of Er^III^ with the amino groups, as well as their co-existence without mutual neutralization has been clearly demonstrated by the higher efficiency of the bifuctional catalyst (entry 1, Table 2) compared to MCM-Er or MCM-NH_2_ (entries 3–5, Table 2) used alone or as physical mixture. In particular a controlled the conjugate product **1** was obtained by Henry reaction while a controlled reaction, giving rise the aldol adduct **2a**, was observed in aldol condensation. The reason of this different behavior is probably the relatively weakness of the base site, able to induce the final dehydratation step only on the more acidic proton. While the reactions did not be activated by acid catalysis (entry 2, Table 2), the formation of a side-product was observed in the Henry reaction when the base catalyst was employed (entry 3, Table 2). The low yields registered when an equimolar amount of MCM–Er and MCM–NH_2_ as a physical mixture was used as catalyst (entry 4, Table 2) confirmed the hypothesis that the proximity between the two active sites on the same support has a pivotal role on the catalytic activity. Moreover an additional test was realized performing both the Henry and the aldolic reactions in the presence of a homogeneous mixture of propylamine and ErCl_3_ (entry 5, Table 2). As shown in Table 2, such a mixture is only slightly active, probably because of the self–quenching occurring between the two functionalities when they are used in the same reaction bulk without immobilization on a solid support.

**Table 2:** Catalytic activity.

Entry	Catalyst ^a^	Henry Reaction (1)				Aldol Reaction (2a)			
		Yield (%)	TON ^b^	TOF ^b^			Yield (%)	TON ^b^	TOF ^b^
**1**	Er-Cys05	62	3.5	0.17			90	6.7	0.39
		59 ^c^	4.41	0.22			87 ^c^	6.3	0.38
		57 ^d^	3.7	0.18			82 ^d^	4.8	0.28
**2**	MCM-Er	16	1.0	0.05			–	–	–
**3**	MCM-NH_2_	10 ^e^	0.6	0.03			43	3.1	0.18
**4**	MCM-Er/MCM-NH_2_	25 ^f^	1.5	0.07			30	2.1	0.12
**5**	Propylamine/ErCl_3_	10	0.6	0.03			25	1.8	0.10

^a^ 10% mol of catalyst was used. ^b^ TON = mmol product/mmol catalytic sites; TOF = mmol product/mmol catalytic sites × hours. ^c^ run II yield after acid wash. ^d^ run III yield after acid wash. ^e^ The side-Michael adduct was recovered in 70% yield. ^f^ The side-Michael adduct was recovered in 25% yield.

It has been demonstrated that the Henry reaction rate determining step is the formation of an imine intermediate between the aldehyde and the amino group on the catalyst [30]: a Lewis acid placed in an appropriate spatial arrangement could play a role of co-catalyst for the imine formation and hydrolysis after the nucleophilic attack. This is congruent with the higher activity displayed by Er-Cys05 (entry 1, Table 2) compared to MCM-Er or MCM-NH_2_ (entries 2 and 3, Table 2). Moreover, the different reactivity between the Henry and aldolic condensation reactions is partially due to the dimension of the substrate that has to react with the amine group.

**Figure 3:**
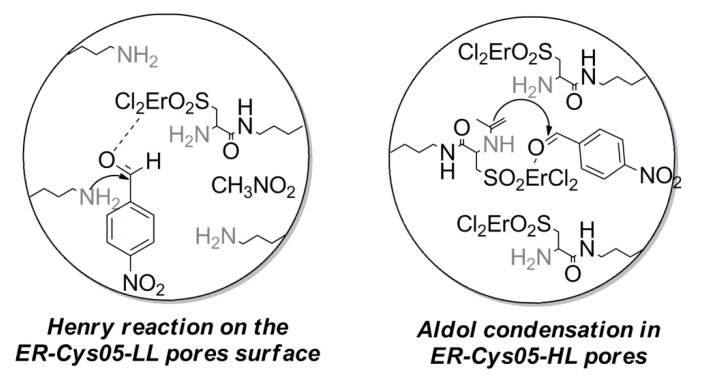
Proposed reaction mechanisms: (**Left**) Henry reaction catalyzed by Er-Cys05; (**Right**) aldol condensation catalyzed by Er-Cys05.

We supposed that the reaction was activated by a mutual cooperation of the two active sites: on the one hand, the amino groups interact with the *p*-nitrobenzaldheyde and acetone, respectively, to induce the enamine formation in the Henry and the aldol condensation, and, on the other hand, the Lewis acid Er^III^ coordinates the carbonyl group of the aldehyde (Figure 3). The more difficult diffusion through the pores of Er-Cys05 experimented by a bigger molecule, such as *p*-nitrobenzaldheyde, compared to acetone, could be the reason of the lower catalytic activity in the Henry reaction. Interestinglythe aldol condensation was selective for the aldol adduct and no H_2_O elimination and consequent formation of the α,β-unsatured carbonyl compound was observed with Er-Cys05 (Scheme 2). Noteworthily, we registered a good conversion and selectivity with a primary amine, comparable to those reported in the literature for secondary or tertiary amines [4,13,28].

A significant loss of activity was registered when the catalyst was recovered after simple solvent washing. Probably due to a progressive pore occlusion with the probable consequence of the inaccessibility of active sites (data not shown) [27]. For this reason recovery of the catalyst was realized by washing with an acid solution by HCl 3N able, in principle, to separate amino groups interacting through hydrogen bonds. The acid wash was then followed by a basic wash with a saturated solution of NaHCO_3_ in order to neutralize the protonation occurring on the amino groups during the acid treatment. Yields registered after three reaction runs followed by this acid/base treatment (entries 3 and 7, Table 2) showed only a slight a loss of catalytic activity. This result was in agreement with the slight metal leaching measured after each run by ICP–MS (see Supplementary Materials).

## 3. Experimental

### 3.1. General Information

ICP-MS measures were performed in a quadrupole-based ICP–MS system XSERIES 2 ICP-MS, from Thermo Fisher Scientific (Thermo TR, Waltham, MA, USA), working in standard mode. The element concentration was determined against external calibration using a synthetic acid multielement calibration standard (IV-ICPMS-71A Inorganic VENTURES, Christiansburg, VA, USA). UV-Vis quantification of amino groups was performed using a Perkin– Elmer UV/VIS Spectometer Lambda 35 connected to a Perkin Elmer UV WinLab acquisition software (Perkin– Elmer GmbH, Überlingen, Germany). BET surface area and physical properties of samples were evaluated by N_2_ adsorption/desorption isotherms carried out at 77 K on a Micromeritics ASAP 2020 sorption analyzer (Peschiera Borromeo, Milano, Italy). The specific surface area was determined applying the BET equation to the isotherm. Mesopore size distribution was calculated using the adsorption branch of the nitrogen adsorption isotherm and the Barrett-Joyner-Halenda (BJH) formula. The average pore diameter and the cumulative volume were obtained from the distribution curve of the mesopore sizes. FT-IR spectra were recorded on a Jasco 430 instrument (Milano, Italy). ^13^C-HRMAS NMR spectra was recorded on a Bruker Avance 500 MHz instrument (Milano, Italy) at 298 K. Chemical shifts are given in parts per million (ppm) from tetramethylsilane as the internal standard (0.0 ppm). Coupling constants (*J*) are given in Hertz. MW–assisted grafting reactions were performed in Synthos 3000 instrument from Anton Paar (Torino, Italy), equipped with a XF-100 and an optical fiber probe as internal control of the temperature. C-C bond formation reactions were monitored by TLC using silica plates 60-F264 on alumina, commercially available from Merck (Vimodrone, Milano, Italy). Liquid Flash chromatography was performed on a VERSA FLASH HTFP station (Supelco, distributed by Sigma-Aldrich) on silica cartridges commercially available from Supelco. All solvents were distilled before using by standard methods. All chemicals were used as commercially available.

### 3.2. Synthesis of Er-Cys05

MCM-41 (5 g) was treated with an aqueous solution of HCl 25% v/v (125 mL) for 3 h at reflux temperature. The temperature was then lowered to room temperature, the mixture was filtered and the solid was washed with water and dried overnight at 90 °C. Pre–treated MCM–41 (1.25 gr) was reacted, in dry toluene (25 mL), with an excess of aminopropyltriethoxysilane (APTES) (12.5 mL) as silylating agent, in a sealed teflon vessel of an Anton-Paar Synthos 3000 MW–oven equipped with a magnetic stirrer previously dried overnight at 90 °C. The temperature was fixed at 130 °C and it was continuously controlled by an internal optical fiber controller for 10 min. At the end of the reaction the system was cooled down to room temperature. The solid was filtered, washed three times with THF and extracted for 2 h in CH_2_Cl_2_/Et_2_O mixture using a Soxhlet extractor, then dried under vacuum and stored overnight at 70 °C. The obtained functionalized mesoporous material (1.50 gr, 1 mmol/gr. silica) was used for the coupling with N-Fmoc-S-Trityl-l-cysteine (5 eq). The aminoacid was solubilized in dry DCM (20 mL) in a two neck round bottom flask equipped with a magnetic stirrer, under N_2_ atmosphere. 5 eq of HOBt, previously dried overnight at 70 °C, were added together with the appropriate amount of DMF, until obtaining of a limpid solution. 5 eq of DIC were then drop by drop added to the solution and the mixture was stirred for 20 min before adding the mesoporous functionalized silica. The suspension was left under gentle stirring for 72 h. After this time the silica was filtered, washed with DCM and the procedure was repeated three times using fresh reactants. Once the reaction ended, the solid was filtered, washed three times with DCM and extracted for 2 h in CH_2_Cl_2_/Et_2_O mixture using a Soxhlet extractor, then dried under vacuum and stored overnight at 70 °C. The resin was then swelled with DCM in a fritted filter funnel equipped with a glass cup. After swelling, the solvent was filtered off and the funnel was refilled with 10 mL/gr silica of a DCM/TIS/TFA (94:5:1) solution, then capped and left under mechanical stirring for 2 min. The solvent was filtered off under N_2_ pressure and the procedure was repeated three times before washing the silica with DCM, extracting it for 2 h in CH_2_Cl_2_/Et_2_O mixture using a Soxhlet extractor, then drying under vacuum and storing overnight at 70 °C. The thiolic moiety on the resin was oxidized with H_2_O_2_ 30% (excess) for 24 h at room temperature. After filtration and washing with water (30 mL × 3) and ethanol (30 mL × 3), solid material was treated with ErCl_3_ (2 eq) in acetonitrile (10 mL) at reflux temperature for 24 h. After cooling the sample up to room temperature the mixture was filtered, washed with acetonitrile, extracted for 2 h in CH_2_Cl_2_/Et_2_O mixture using a Soxhlet extractor, then dried under vacuum and finally kept at 70 °C overnight. 1 gr of the silica was finally suspended in DMF (200 mL) and mechanically stirred for 2 h before adding 4 mL of DBU (2% v/v solution). The suspension was stirred for 2 h more, then the silica was filtered, washed with DCM (30 mL × 3) and Et_2_O (30 mL × 3) and extracted for 2 h in CH_2_Cl_2_/Et_2_O mixture using a Soxhlet extractor. The solvent was evaporated under vacuum and, after drying at 70 °C overnight, the resulting bifunctional catalyst was stored under dried conditions.

[-NH_2_] loading: (mmol/gr silica): 0,94 [Er^III^] loading: (mmol/gr silica): 0,94. Specific surface area BET: 327 m^2^/gr; Pore Volume (BJH): 0.24 cm^3^/gr; Pore diameter (BJH): 40 Å. FT-IR ν=: 465 (s, *νs* (Si-OSi)), 791 (m, *νs* (C-S)), 1312 (w, νs (S=O)), 1550 (w, δ (N-H/C-N)), 1650 (s, *νs* (C=O)), 2342 (w, *νs* (N-H_3_^+^)), 3442 (*vs*, (HO-H)) cm^−1^.

### 3.3. Aldol Reaction

In a typical procedure, to a solution of *p*-nitrobenzaldehyde (1 mmol) in dry acetone (20 mL), placed in a round bottom flask equipped with condenser and magnetic stirrer, 10% mol of bifuctional catalyst was added under inert atmosphere. The mixture was heated in an oil bath at 50 °C for 17 h and the reaction was monitored by TLC until disappearance of the aldehyde. The reaction was cooled, the silica was filtered off and washed with CHCl_3_ (3 × 20 mL) and acetone (3 × 20 mL). The solution was then evaporated under reduced pressure and the resulting reaction crude was purified by liquid flash chromatography (petroleum Ether/EtOAc 6:4 v/v as eluent) and the yield determined on the pure product. After evaporation under reduced pressure the catalyst was suspended in 3N HCl and stirred at room temperature for 3 h, washed with water (×3), followed by a satured solution of NaHCO_3_ (×3) and again water until neutralization. The solid was then filter off, washed with water and dryed at 70 °C overnight. The catalyst was stored under dry atmosphere and reused in a new reaction cycle.

### 3.4. Henry Reaction

In a typical procedure, to a solution of *p*–nitrobenzaldehyde (1 mmol) in nitromethane (20 mL), pplaced in a round bottom flask equipped with condenser and magnetic stirrer, 10% mol of bifuctional catalyst was added. The mixture was heated in an oil bath at 50 °C for 20 h and the reaction was monitored by TLC until aldehyde disappearance. The reaction was cooled, the silica was filtered off and washed with CHCl_3_ (3 × 20 mL) and acetone (3 × 20 mL). The solution was then evaporated under reduced pressure and the resulting reaction crude was purified by liquid flash chromatography (dichloromethane/MeOH 98:2 v/v as eluent) and the yield determined on the pure product. After evaporation under reduced pressure the catalyst was suspended in HCl 3 N and stirred at room temperature for 3 h, washed with water (×3), followed by a saturated solution of NaHCO_3_ (×3) and again water until neutralization. The solid was then filter off, washed with water and dryed at 70 °C overnight. The catalyst was stored under dry atmosphere and reused in a new reaction cycle.

## 4. Conclusions

We have described a method to successful synthesize two new bifuncional Er^III^-based metallorganic heterogeneous catalysts by MW-assisted grafting using the natural aminoacid l-cysteine, after appropriate modification of the lateral chain, as ligand for Er^III^ and as amino groups source. The correct spatial arrangement of acid/base sites allowing cooperation and their peaceful co-existence on the silica surface has been demonstrated. The catalysts showed a good catalytic activity for C-C bond formation in the Henry reaction and aldol condensation. The catalyst was recovered, reactivated by acid/base wash and reused without a significant loss of activity.

## Supplementary Materials

Supplementary materials can be accessed at: http://www.mdpi.com/1420-3049/19/7/10218/s1.
