# Supplementary material for: An Erbium-Based Bifuctional Heterogeneous Catalyst: A Cooperative Route Towards C-C Bond Formation
Source: Molecules. 2014 Jul 15;19(7):10218–29. doi: 10.3390/molecules190710218 (PMC6271265; doi:10.3390/molecules190710218)
Supplement: Supplementary file 1 [file molecules-19-10218-s001.pdf]

Supplementary Materials

1. Silica Amino Loading by Spectrophotometric Analysis of the Fmoc-Protecting Groups

1.1. Fmoc Derivatization of Amino Groups

A small amount of resin Cys01 was suspended in a $NaHCO_3$ solution (0.2 M). At this mixture was added a freshly prepared Fmoc-Cl solution in methanol (50 mM). The resulting dispersion was stirred under mechanical agitation for 30 min. After this time the suspension was filtered, and then washed with H_2O-H_2O/THF-THF-Et_2O, the solvent was removed under reduced pressure and the resin was dried at 70 ℃ overnight.

1.2. Fmoc-Estimation by UV Analysis

The loading was evaluated by photometric determination of the Fmoc chromophore leaved upon treatment with DBU/DMF [1]. Dry Fmoc amino resin was weighed into a 10 mL graduated flask. The resin suspended in DMF (2 mL) was mechanical stirred for 30 min. DBU (40 μL) was added to the mixture in order to obtain a 2% basic solution, then the resulting dispersion was gently agitated for 30 min. The mixture was diluted to 10 mL with MeCN. 2 mL of this solution was diluted in a 25 mL graduated flask with MeCN. A blank was prepared at the same procedure without addition of the resin. The solution was analyzed in a UV/VIS Spectrophotometer and the absorbance recorded at λ = 304 nm. The analysis was repeated three times and the loading was calculated using the equation below:

$$Loading\ \ (mmol/g) = \frac{\bar{A}\ \times 16.4}{mg\ resin} \tag{1}$$

The results of Fmoc-estimation analysis are summarized in Table S1.

2. ICP/MS Determination of Er^{III}

In order to determine the Er^{III} loaded on the silica surface or leached during reactions, a MW-assisted digestion procedure was applied to the functionalized silica or the reaction solutions. Silica loaded with erbium(III) (~30 mg) or reaction solutions (1 mL) were digested with 8 mL Suprapure HNO_3 (65%, v/v, Merck) using an Anton-Paar Multiwave 3000 microwave digester, equipped with a XF100 rotor (operating pressure: 60 bar). One randomly selected vessel was filled only with reagents and used as a blank. Digestion was conducted in “power controlled” mode. After digestion, the vessels have been cooled down, the digests were filtered with a single use filter unit (0.20 μm), then diluted to 50 mL using purity water (obtained from a Milli-Q water purification system, Merk Millipore, Darmstadt, Germany).

ICP-MS measurements were performed in a quadrupole-based ICP-MS system XSERIES 2 ICP-MS, from Thermo Fisher Scientific, working in standard mode. Samples were introduced in a quartz concentric nebulizer by a peristaltic pump (selected speed of 30 rpm). The element concentration was determined against external calibration using a synthetic acid multielement calibration standard (IV-ICPMS- 71A Inorganic VENTURES).

Table S1. The [-NH_2] and the Er^{III} loadings for all the intermediates of the catalyst synthesis.

Entry	Intermediate	Er-Cys05		
		Total [-NH_2] (mmol/gr)	AA [-NH_2] (mmol/gr)	[Er^{III}] (mmol/gr)
**1**	Cys 01	1.00	1.00	-
**2**	Cys 02	1.00	1.00	-
**3**	Cys 03	0.94	0.94	-
**4**	Er-Cys 04	0.94	0.94	0.94
**5**	Er-Cys 05	0.94	0.94	0.94

3. Leaching Studies

In order to evaluate the possibility to recover and reuse the catalyst, a recycle test and the Er^{III} leaching ICP-MS measurement were performed on Er-Cys05. The results of Er^{III} leaching of the recycled resin are summarized in Table S2.

Table S2. Recycle Tests.

Entry	Run	Er^{III} leaching (%) [a]
**1**	1	-
**2**	2	0.25
**3**	3	0.04

4. FT-IR Spectra

MCM-41 Sample

Figure S1. FT_IR spectrum of MCM-41 starting material.

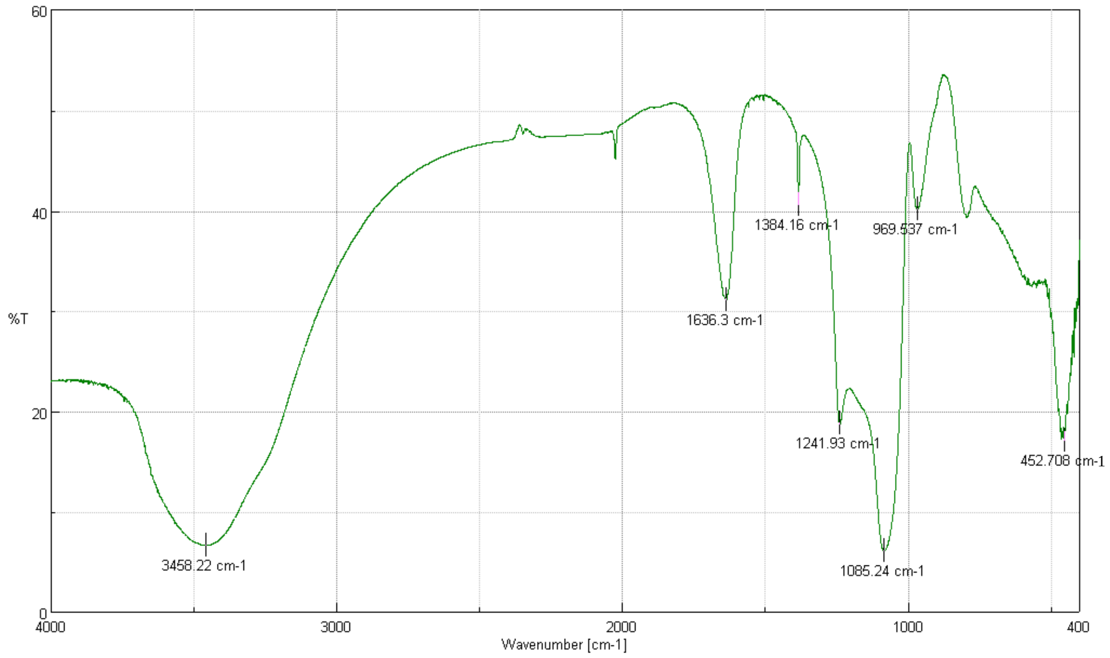

Cys-01 Sample

Figure S2. FT_IR spectrum of Cys-01 intermediate.

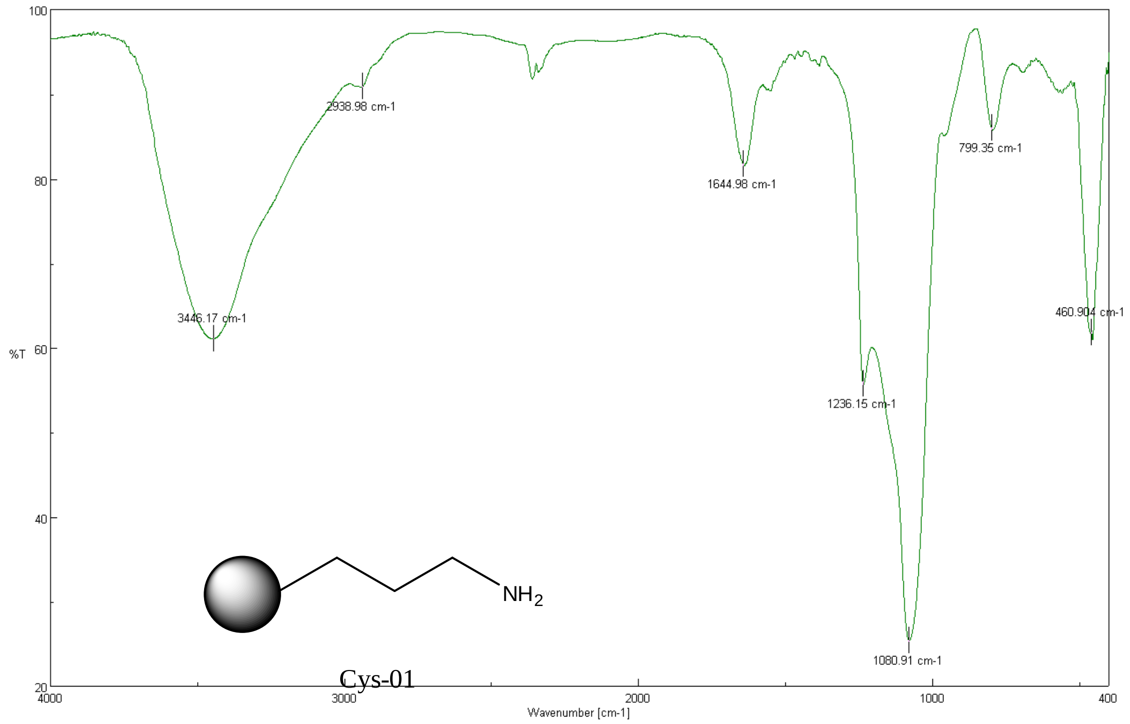

Cys-02 Sample

Figure S3. FT_IR spectrum of Cys-02 intermediate.

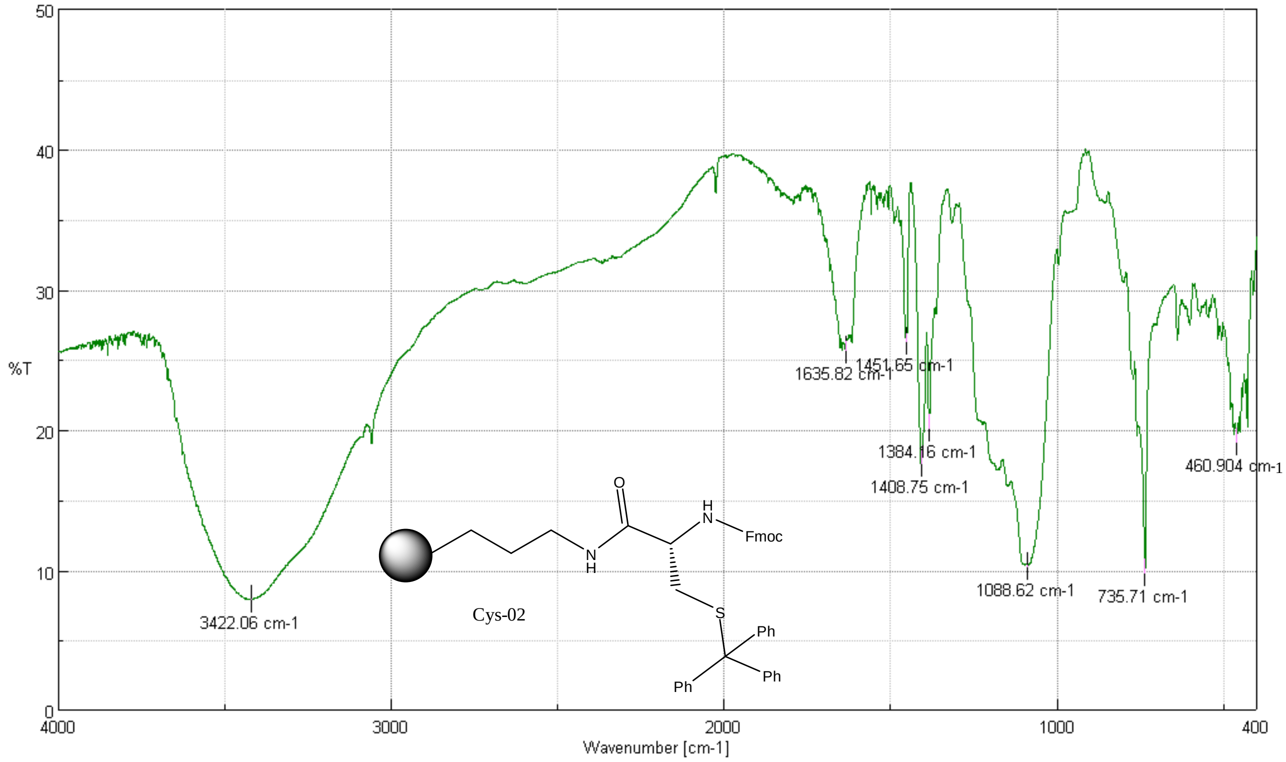

Er-Cys-05 Sample

Figure S4. FT_IR spectrum of Er-Cys-05 catalyst.

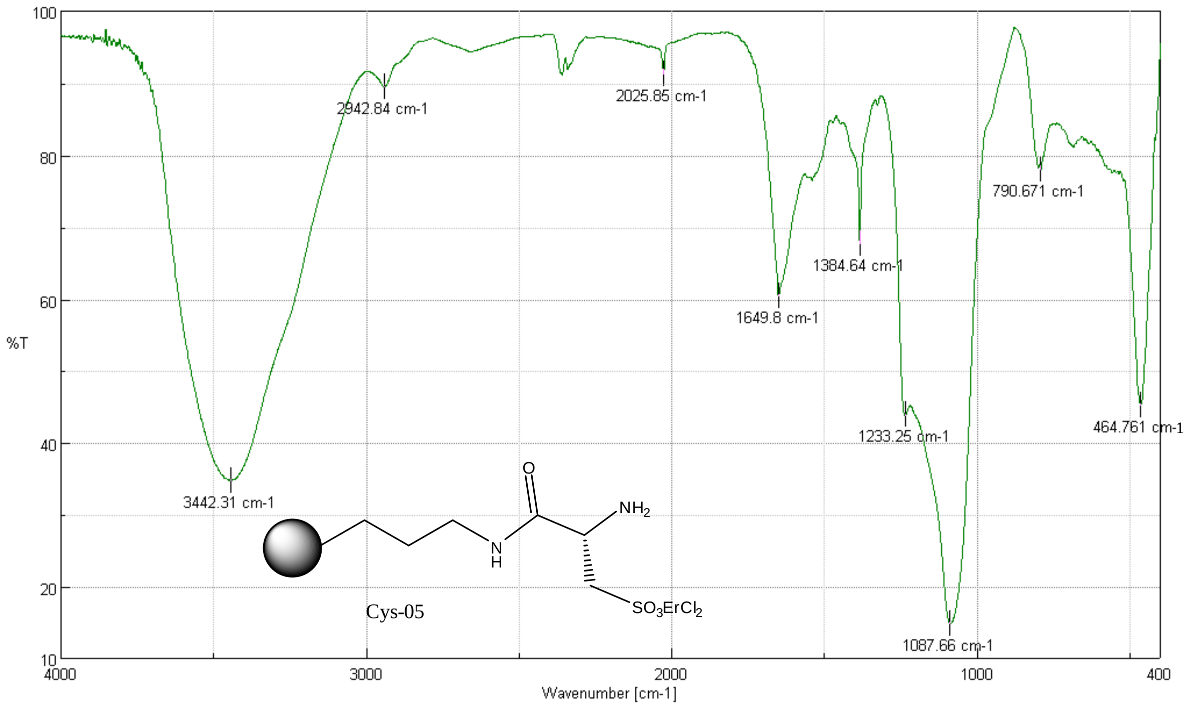

5. ^{13}C-NMR Spectra

Cys-01 Sample

Figure S5. ^{13}C-NMR spectrum of Cys-01 intermediate.

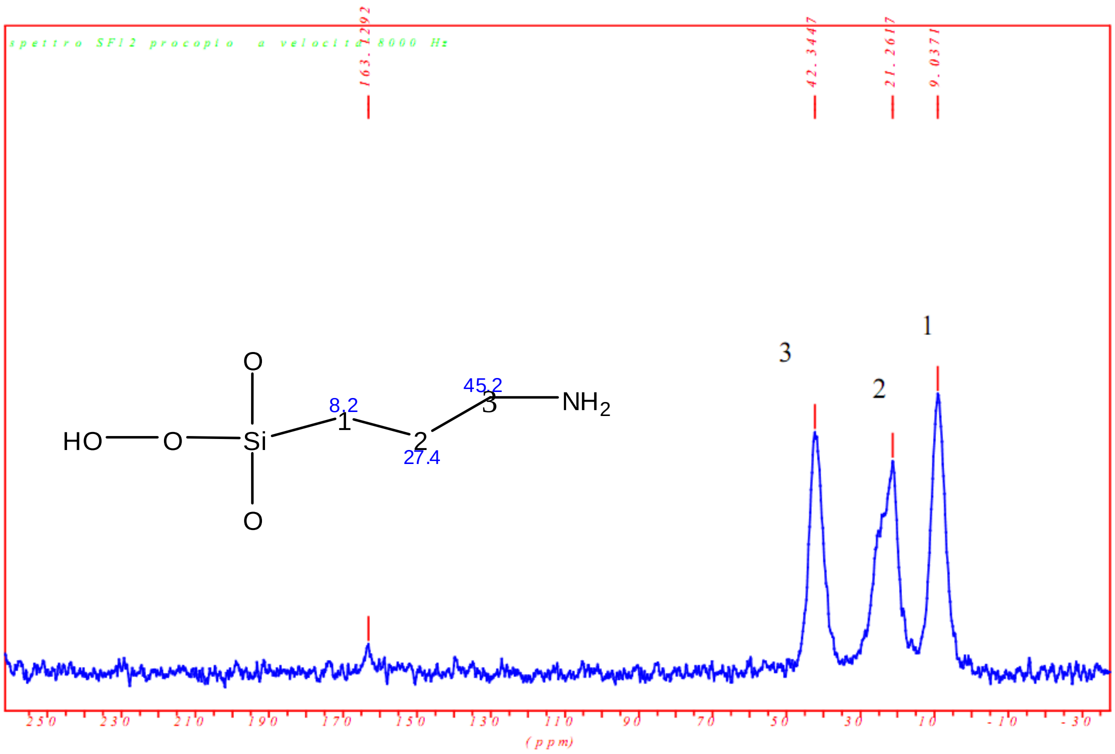

Cys-02 Sample

Figure S6. ^{13}C-NMR spectrum of Cys-02 intermediate.

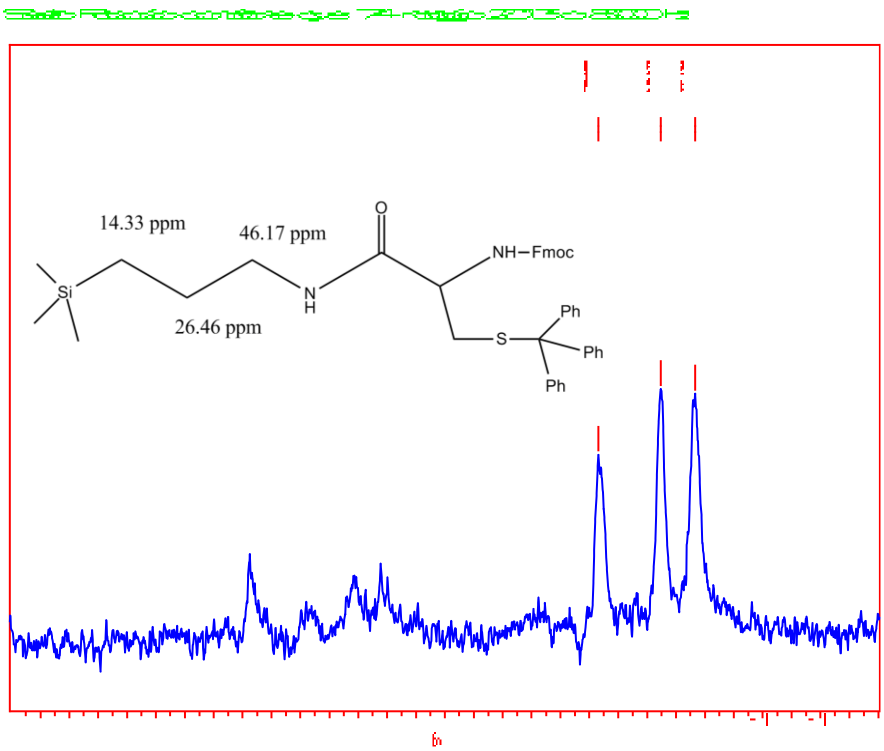

Cys-03 Sample

Figure S7. ^{13}C-NMR spectrum of Cys-03 intermediate.

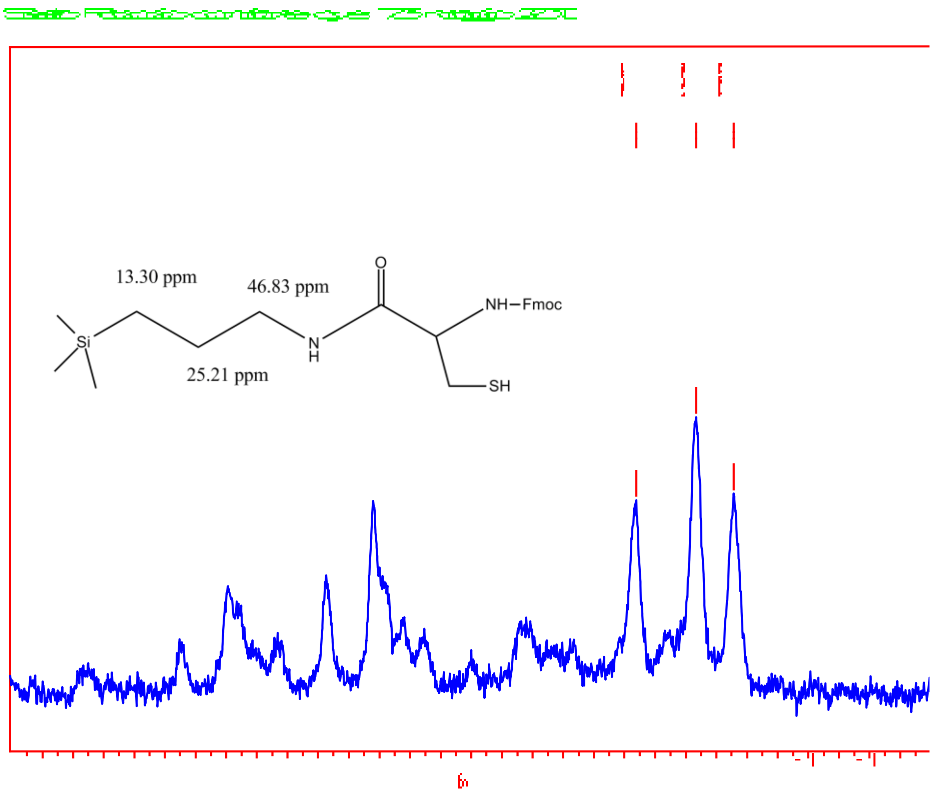

6. N_2 Adsorption/Desorption Curves

Isotherm Linear Plot of MCM-41 vs Er-Cys05

Figure S8. MCM-41 *vs.* Er-Cys05 textural properties by N_2 Adsorption/Desorption.

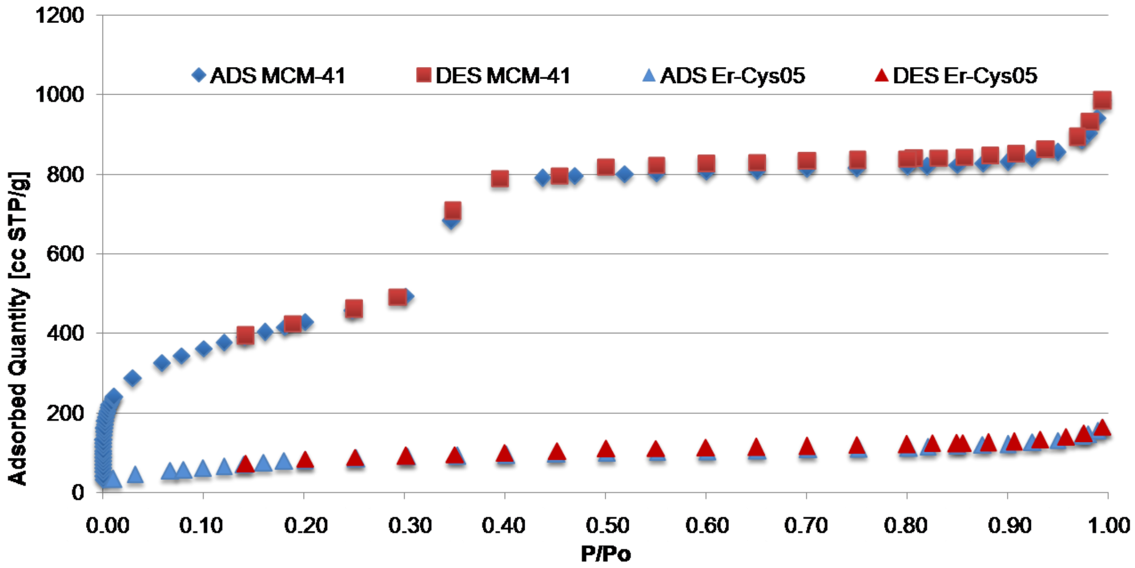

Catalyst	S BET (m^2/gr)	Pore Volume (cm^3/gr)	Average Pore Diameter (Å)
**MCM-41**	1600	1.70	35
**Er-Cys05**	330	0.24	40

7. BJH Pore Diameter Distribution

MCM-41

Figure S9. Linear Plot of MCM-41 BJH Pore Diameter Distribution.

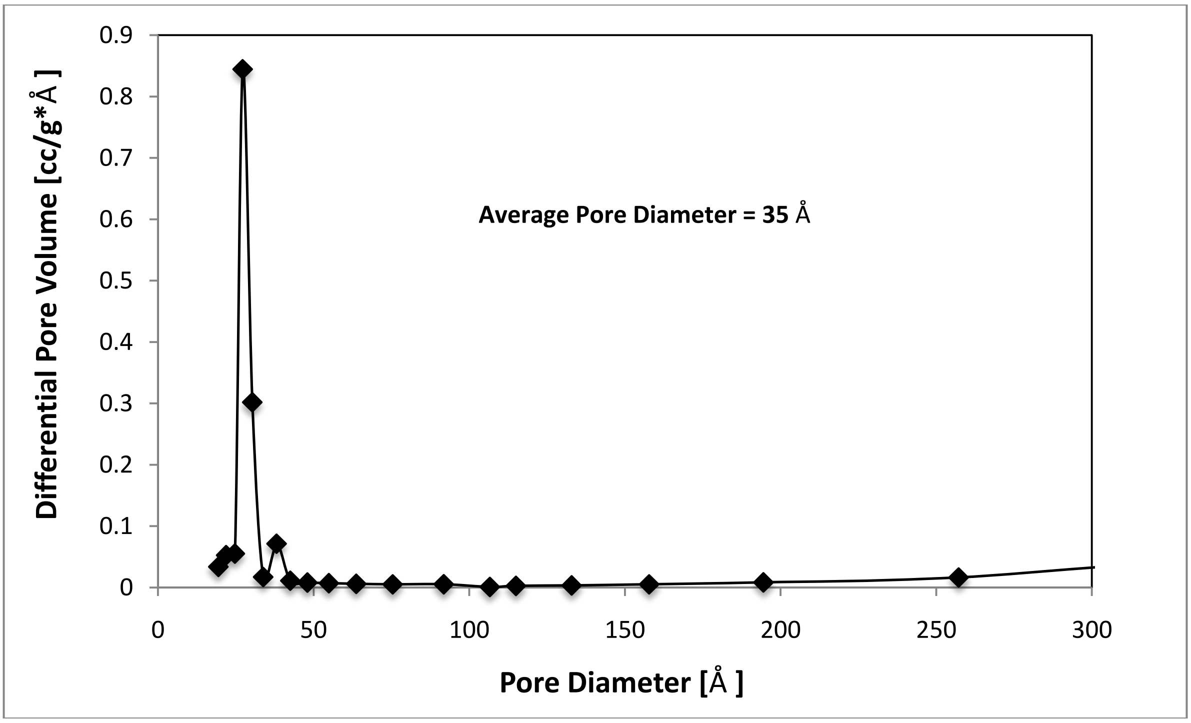

Er-Cys 05

Figure S10. Linear Plot of Er-Cys-05 BJH Pore Diameter Distribution.

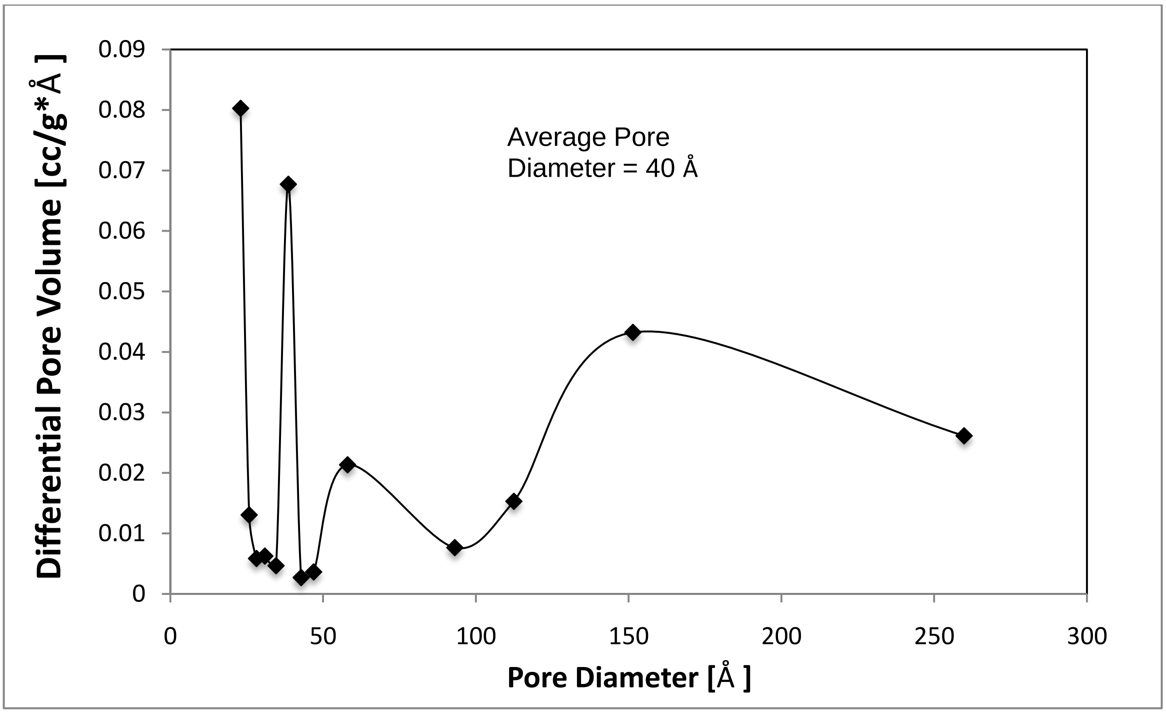

8. Comments to N_2 Adsorption/Desorption Curves

Characteristics of catalysts surface were monitored by N_2 adsorption-desorption technique. As expected the catalyst, after loading of active sites, shows a lower specific surface area with respect to the starting MCM-41 support (Figure S8), indicating a large grafting of the support surface. Particularly, the typical isotherm of mesoporous materials (isotherm of type IV) showed by the MCM-41 support drastically changes after grafting: the isotherms of Er-Cys05 catalysts do not show the same path of the starting support, especially regarding the "meniscus curve" (P/Po = 0.3/0.4) that, generally, is related to the condensation of the gas [2,3]. The relative pressure at which the condensation occurs is strictly correlated to the pore size of the porous solid. Particularly, when the meniscus curve occurs at a well-defined relative pressure value, the pore size distribution is very narrow [2,3]. After grafting, the N_2 adsorption isotherm of Er-Cys05 material shows that this catalyst preserves a slight regular mesoporosity, even if the meniscus part of the curve is broader and occurs at lower relative pressure with respect to the isotherm of the starting support. Both pore size distributions are derived from N_2 adsorption isotherms at 77 K, of corresponding material, and according to the BJH model [4] The average pore diameter of all calcined MCM-41 materials is c.a. 35 Å, with a large contribution of pores having 27 Å of dimension, while the pore distribution of final catalyst shows different pore size contribution due to the post-synthesis treatments. In any case the average pore diameter is c.a. 40 Å.

References

1. Molnár-Perl, I. *Quantitation of Amino Acids and Amines by Chromatography: Methods and Protocols*; Elsevier: Amsterdam, The Netherlands, 2005; p. 476.

2. Rouquerol, F.; Rouquerol, J.; Sing, K. *Adsorption by Powders and Porous Solids, Principles, Methodology and Applications*; Elsevier Ltd.: Amsterdam, The Netherlands, 1999.
3. Bond, G.C. *Heterogeneous Catalysis, Principles and Applications*, 2nd ed.; Oxford University Press: Oxford, UK, 1987.
4. Groen, J.C.; Peffer, L.A.A.; Perez-Ramirez, J. Pore size determination in modified micro- and mesoporous materials. Pitfalls and limitations in gas adsorption data analysis. *Micropor. Mesopor. Mat*. **2003**, *60*, 1–17.
